# Statistical Fragility and Methodological Constraints in Metabolomic Mediation of Air Pollution and Cardiovascular Risk

**DOI:** 10.1016/j.jacadv.2026.103150

**Published:** 2026-08-13

**Authors:** Man-Ju Ting

**Affiliations:** Institute of Environmental and Occupational Health Sciences, College of Public Health, National Taiwan University, Taipei, Taiwan

We read with interest the report by Liu et al^1^ regarding the integration of air pollution exposure, untargeted metabolomics, and major adverse cardiovascular events (MACE) in patients undergoing cardiac catheterization. Although the study offers an innovative look into environmental exposomics, several methodological, and statistical constraints materially limit the strength of its mechanistic inferences.

First, the study appears to overstate mediation in the setting of a weak primary epidemiologic signal. The central narrative implies that nitrogen oxides (NOx) drive cardiovascular risk through specific metabolic pathways, particularly choline metabolism. However, the reported association between NOx and incident MACE did not reach conventional statistical significance (subdistribution HR: 1.19; 95% CI: 0.99-1.44; *P* = 0.068), whereas PM_2.5_ was null for the composite endpoint. Mediation analysis cannot compensate for an uncertain exposure-outcome relationship; rather, it risks converting statistical fragility into mechanistic overinterpretation.^2^

Second, the temporal architecture is poorly aligned with causal mediation. Air pollution exposure was estimated based on the year preceding enrollment, whereas the plasma metabolome is highly dynamic and sensitive to short-term physiologic perturbation, recent diet, and acute illness. In a cohort undergoing cardiac catheterization, metabolic profiles likely reflect periprocedural stress or active ischemia rather than stable intermediates of chronic pollution exposure. Furthermore, using residence-based modeling at enrollment to predict outcomes over a 9.8-year follow-up period ignores residential mobility and changes in local traffic patterns, leading to potential exposure misclassification.

Third, the pivotal role of choline as a mediator warrants caution regarding dietary confounding. Choline and its metabolites are highly sensitive to dietary intake and gut microbiome composition factors that often cluster with socioeconomic status and urban environments.^3^ Without adjusting for these, the NOx-choline-MACE pathway may simply be a surrogate for unmeasured lifestyle patterns. In addition, the study's reliance on a catheterization-referred cohort introduces collider bias; conditioning on clinical referral can distort the associations between exposures, metabolites, and outcomes, threatening internal validity.^4^

Finally, the analytical burden appears disproportionate to the sample size. Analyzing thousands of metabolic features in 244 participants with only 97 MACE events creates substantial risk for overfitting and stochastic associations. Although false discovery rate corrections were applied, the lack of external validation or targeted quantitative assays suggests these findings remain hypothesis-generating.

In conclusion, Liu et al^1^ provide a valuable exploratory signal, but larger studies with repeated biospecimen collection and rigorous handling of selection mechanisms are required before these metabolic signatures can be viewed as definitive mediators of environmental risk.
